# A Technical Note on a Modified Subcrestal Buccal Flap Approach to All-on-Four: A Case Report

**DOI:** 10.7759/cureus.58453

**Published:** 2024-04-17

**Authors:** Fernando Giovanella, Fábio Bezerra, Michel A Dantas Soares, Guilherme Oliveira, Bruna Ghiraldini

**Affiliations:** 1 Dentistry, Private Practice, Blumenau, BRA; 2 Biotechnology, Universidade Estadual Paulista (UNESP), São Paulo, BRA; 3 Engineering, S.I.N. Implant System, São Paulo, BRA; 4 Periodontics, Universidade Federal de Uberlândia (UFU), Uberlândia, BRA; 5 Implantodontology, Universidade Paulista (UNIP), São Paulo, BRA

**Keywords:** osseointegration, full arch rehabilitation, dental implants, atriphic ridge, all on four

## Abstract

This case report describes a dual full-arch rehabilitation focusing on a modified buccal incision for installation of four implants for full-arch rehabilitation of an edentulous maxilla. A modified buccal incision was performed in the subcrestal buccal region to promote direct access to the periosteum without incising the muscles in the region. For the installation of anterior implants, an 8.5 mm implant was locked in the cortical bone of the alveolar ridge and in the cortical bone of the floor of the pyriform cavity. The drilling point of the posterior implants was defined using the anterior implants as a visual reference, and the entry point could be visually estimated from the topography of the palatal surface of the maxilla. After bone leveling, the drilling enlargement sequence was carried out using drills that allowed the installation of long implants (18 mm). Straight mini-abutments were installed in the anterior implants and angled at 30º in the posterior implants. The flap was then perforated in the exact region where the mini-abutments were located. The buccal incision line was sutured with continuous 5-0 nylon suture. On the following day, aesthetic tests were carried out with teeth mounting. The patient presented minimal edema, and the lip motricity and smile width were completely preserved. The prosthesis was delivered five days after surgery. The suture was removed, and the prosthesis was installed while maintaining compression on the gingival tissue. The patient reported no pain during the prosthesis installation. The modified buccal flap enables implant placement for full-arch rehabilitation of an edentulous maxilla.

## Introduction

Atrophic jaws are a challenge in oral rehabilitation using prostheses supported by dental implants, since the extensive reabsorption of the alveolar process, which occurs due to a large amount of edentulous time, may restrict the use of conventional implants as a result of limitations in the amount of bone tissue, hindering implant placement in an appropriate position [1]. To manage this type of clinical condition, guided bone regeneration and maxillary sinus floor elevation techniques have been indicated, however, these surgical procedures increase the time, cost, and morbidity of rehabilitative treatment [1-3].

The conventional surgical protocol for placing full-arch fixed prostheses in the jaws involves installing six to eight implants. However, the all-on-four technique has been described as an alternative for full-arch rehabilitation, with excellent cost-benefit when compared to the conventional protocol of implants installed axially, using inclined implants in the pre-existing bone to avoid bone grafting surgeries [4,5]. The all-on-four technique consists of installing four implants in the anterior region of the maxilla, with the two most anterior implants installed axially, seeking primary stability in the cortical bone of the base of the nasal fossa and two posterior implants installed in an angled manner distally, touching the maxillary sinus and minimizing the cantilever, achieving primary locking in the cortical bone of the canine pillar or in the zygomatic bones or pterygoid process of the sphenoid bone [5-7]. To obtain good implant locking using the all-on-four technique in atrophic maxillary conditions, it may be necessary to use long implants that can lock into portions of the basal bone [8,9].

Another approach that can reduce the surgical trauma in full-arch rehabilitation is the use of different flap designs. For the installation of implants in the maxillary residual ridge, the palatal approach has been used, where the implants are installed on the palatal aspect of the residual ridge [5,7]. Total buccal mucoperiosteal detachment is not necessary except to provide visualization of the buccal bone topography, to guide the surgeon to the best location and direction of the drill, avoiding fenestrations. The greater the mucoperiosteal detachment, the greater the formation of edema [10]. Despite being physiological, post-surgical edema can prevent or hinder the precision of the smile tests, in cases of immediate load. In addition, muscle disinsertion in the anterior region of the maxilla can change labial and nasal dynamics [11]. Elevation of mucoperiosteal flaps during implant placement can damage or destroy supraperiosteal vascular branches. For this reason, peri-implant vascularization is greater in implants installed flapless than when a flap is used [12]. Thus, the search for more conservative access routes and maximum maintenance of the adhered periosteum can bring biological benefits to osseointegration.

The anterior region of the bottom of the vestibule sulcus contains salivary glands and the presence of the orbicularis oris muscle. In the intermediate region of the vestibule, there is the insertion of the superior incisive labii muscle, and in the buccal region close to the crest, the gingiva rests only on the periosteum [13]. Therefore, positioning the incision in the subcrestal buccal region promotes direct access to the periosteum without incising the muscles inserted in the region. The present case report describes a modified buccal incision for installation of four implants for full-arch rehabilitation of an edentulous maxilla where the buccal mucoperiosteal and muscle detachment are reduced to a minimum.

## Technical report

A 65-year-old male systemic health patient presenting total edentulism and an atrophic maxilla was treated in the case report. The patient has esthetic and functional complaints about the removable full arch prosthesis. The following case report describes the installation of four implants for full-arch rehabilitation of an edentulous maxilla using long implants that locked apically in the basal bone. A modified buccal incision was performed in the subcrestal buccal region to promote direct access to the periosteum without incising the muscles in the region. This approach to accessing the maxilla reduces the buccal mucoperiosteal and muscle detachment.

**Figure 1:**
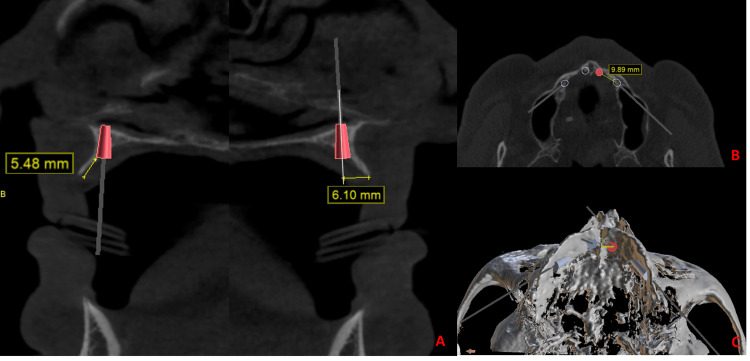
a) Measurement of the amount of bone reduction; definition of the entry point of the initial perforation in the buccopalatal direction in relation to the top of the alveolar ridge; b) Entry point of one of the distal implants – axial view; c) Entry point of one of the distal implants – 3D view

For the installation of anterior implants, vertical bone availability was initially defined, after vertical bone reduction. A bone reduction of approximately 5.5 mm was performed and then an 8.5 mm implant was locked in the cortical bone of the alveolar ridge and in the cortical bone of the floor of the pyriform cavity (Figure 1A). In the anteroposterior direction, the starting point of the perforation was 6 mm from the top of the alveolar crest in the palatal direction. In the lateromedial direction, the center of the incisive canal was the main reference point. The drilling was carried out at 4 mm from the lateral wall of the incisive canal. These coordinates orientate the drilling point for the two previous implants without the need for complete visualization of the buccal portion of the residual ridge (Figure 1B).

The drilling point (location, direction, and angulation) of the posterior implants was defined using the anterior implants as a visual reference, and the entry point could be visually estimated from the topography of the palatal surface of the maxilla. The incision was performed in the buccal subcrestal region, considering the minimum detachment necessary to perform the bone reduction osteotomy. The detachment was carried out in the vestibular region only around 6 mm (to make approximately 5 mm of bone reduction) and the remainder of the periosteum and muscle insertions were completely maintained. The initial drillings were made before bone reduction. In this way, it was possible to have a comparative visual reference with the 3D image of the virtual plan (Figure 1C).

**Figure 2:**
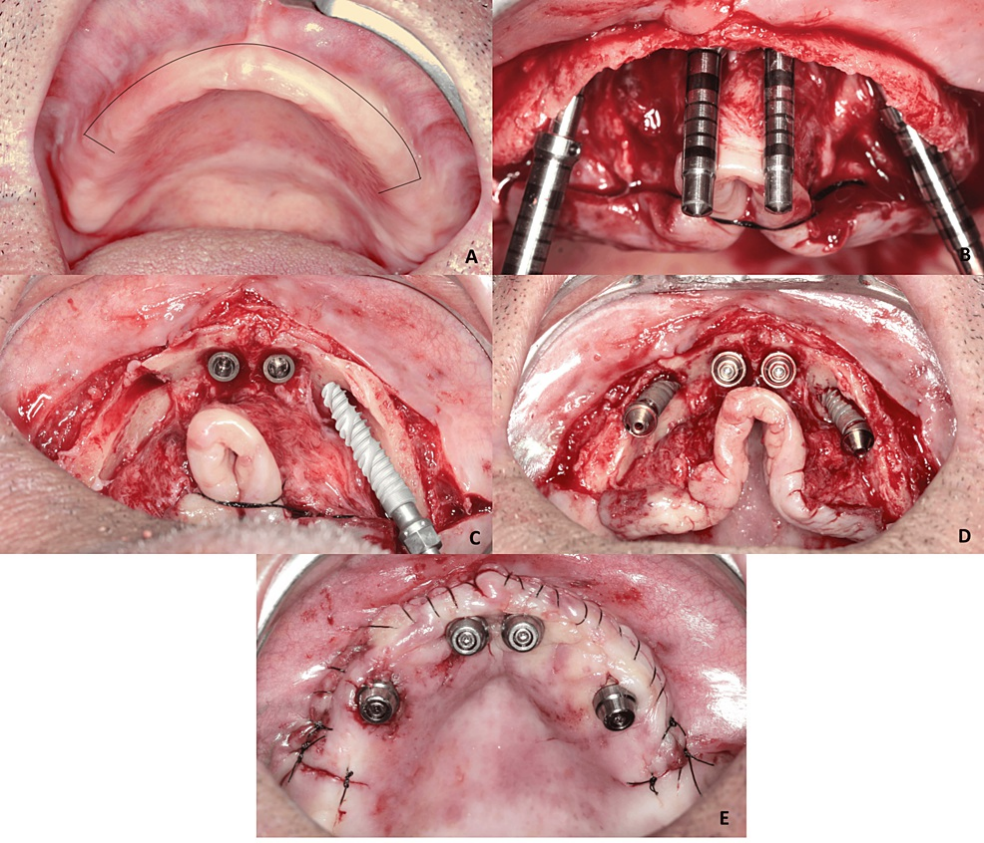
a) Subcrestal buccal incision; b) Conservative buccal detachment just for bone reduction and initial osteotomies performed before bone reduction; c) Posterior implants Epikut Long 18mm - S.I.N. Implant System being installed with total removal of the periosteum only in the regions where the implants will have bone contact. In other regions, the periosteum is kept inserted; d) Installation of mini-abutments; e) Repositioning of the flap and installation of mini-abutment protectors.

On the palatal side, the detachment was performed with emphasis only on the region where the osteotomies were planned to be performed, leaving the maximum amount of periosteum adhered to the surrounding bone. The success of the initial drilling was confirmed by probing the perforation to detect if the entire drilling path was intraosseous. After bone leveling, the drilling enlargement sequence was carried out using drills that allowed the installation of long implants (Epikut Implant; S.I.N. Implant System, São Paulo, Brazil) (Figure 2A-2C).

The complete reflection of the total mucoperiosteal flap was performed vigorously only in the region of the osteotomy for implant insertion. In other areas, the flap was kept adhered to promote a blood supply to the bone and minimize vascular trauma to the bone tissue. All implants obtained an insertion torque > 45Ncm. Subsequently, the straight mini-abutments were installed in the anterior implants and angled at 30º in the posterior implants. The flap was then perforated in the exact region where the mini-abutments were located. The buccal incision line was sutured with continuous 5-0 nylon suture and simple suture in the region of the vertical incisions. The transfer procedure was performed on the recently operated mucosa (Figure 2D, 2E). As a post-operative care, sodium diclofenac was prescribed for three days, dipyrone or paracetamol for three days, amoxicillin for seven days, and mouthwash with chlorhexidine digluconate at 0.12% for 14 days.

**Figure 3:**
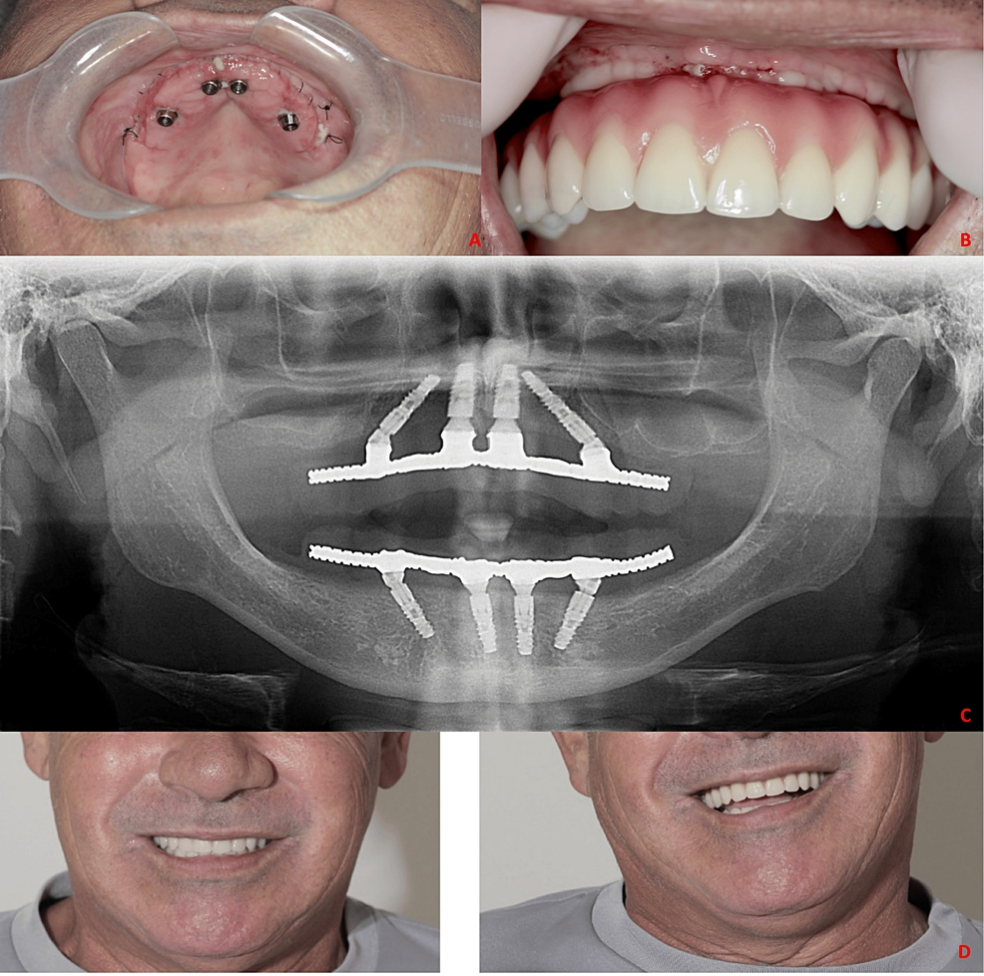
a) Clinical appearance five days after surgery, b) Tissue compression during prosthesis installation; c) Postoperative implant position; d) Clinical aspect of the prosthesis in function.

On the following day, aesthetic tests were carried out with teeth mounting. The patient presented minimal edema. Furthermore, the lip motricity and smile width were completely preserved. The suture was removed, and the prosthesis was installed, maintaining compression on the gingival tissue (Figure 3A). Despite compressive ischemia, the patient reported no pain during the installation of the prosthesis (Figure 3B, 3C). The prosthesis was delivered five days after surgery (Figure 3D).

## Discussion

Although the all-on-four technique has evolved the way that atrophic maxillae are rehabilitated, the type of incision and mucoperiosteal detachment have remained the same as the classical approach for bone reconstructions. Access at the top of the ridge is currently the most recommended approach, with total mucoperiosteal detachment both in the buccal and palatal regions [14,15]. However, with this approach, all musculature in the buccal region is detached and the final suture of the mucosa is performed permeating the mini-abutments.

Surgical access exclusively through the palate can limit the edema and the action of muscular forces on the flaps [16]. However, when performing an incision at the end of the vestibule, invariably a portion of the orbicularis muscle and superior inicivus labii are involved. With a buccal incision close to the top of the ridge, only the mucosa and periosteum are involved [13]. It is also interesting that the inferior border of the incisivus labii superioris (ILS) corresponded to the mucogingival junction in all specimens. This means the ILS could define the border of the keratinized gingiva above the anterior maxillary teeth [17]. Thus, it is possible to perform a small vestibular detachment so that a bone reduction osteotomy can be performed while the musculature remains inserted. It is then possible to optimize the position of the incision and suture line, making them distant from the transmucosal area of the mini-abutments. This approach facilitates the suture, maintains the suture line free of prosthesis compression, facilitates suture hygiene, and makes suture removal easier, and, in some cases, it may even be possible to remove the suture without removing the prosthesis. Another advantage is in relation to the immediate post-operative transfer maneuvers, where the peri-implant gingiva presents an immediate post-operative physical aspect of flapless surgery. This facilitates the installation of the transfers without the interposition of suture threads, such that if acrylic resin leaks in the peri-implant region, the suture will not be broken when removing the impression, in addition to facilitating hemostasis by facilitating intra-oral scanning maneuvers.

Unlike guided surgery, where the drill blades also act on the gingival margin of the transmucosal orifice, promoting tissue damage [18], the possibility of creating the transmucosal orifice after implant placement guarantees a clear cut without bedsores. The transmucosal incision to “dress” the mini-abutments, in the present case, was performed with a conventional scalpel, where a hole compatible with the diameter of the mini-abutment head was created. However, there is the possibility of improving this surgical maneuver with the use of a circular scalpel. A mini linear incision the size of the width of the implant can also be made instead of a circular incision [12,18]. When the circular incision is used, it is noticed that the maintenance of a hermetic seal of the peri-implant soft tissues favors fast healing and improvement in peri-implant health [19]. For this reason, a smaller punch than the diameter of the implant/abutment promotes this sealing. In addition, such an immediate postoperative condition further facilitates the molding process and biological sealing. The ideal maneuver would be to make a 3 mm punch in the mucosa and use a 4 mm wide mini-abutment. Instead of installing the mini-abutment and “dressing” the mucosa over it, the ideal is to install the mini-abutment after repositioning the mucosa with the hole created by the punch in the exact location of the implant. Thus, when the mini-abutment is screwed in, there will be a strong seal between the mucosa and the mini-abutment.

After bone reduction osteotomy, the presence of soft tissue remnants is normal. In the present case, instead of removing this tissue, it was kept and sutured. This made the gingiva extremely “fluffy” so that it could be immediately conditioned with a greater magnitude of compression, but without exceeding the limits of tissue tolerance and pain reference by the patient. Tissue conditioning maneuvers with provisional prostheses improve the emergence profile of the prosthesis base. However, these maneuvers may require several consultations and even months before a good result is achieved. In the present case, due to the maintenance of all the redundant soft tissue, it was possible to carry out an extensive immediate conditioning of the prosthesis, with no report of pain by the patient. In addition, the incision line is outside the direct compression zone. Due to the limited vestibular detachment, the teeth could be tested 24 hours after the surgery, where it was possible to observe the labial dynamics when smiling, with minimal alteration.

The main disadvantage of the access presented is the substantial decrease in the visualization of the maxillary bone topography. Palatal visualization, reference to the incisive canal and tactile perception during drilling are the surgeon's main references. This makes it difficult for less experienced surgeons to perform the technique. Although the definition of the initial drilling site can be extracted from the software, there is a degree of subjectivity, especially for the placement of posterior implants. However, if perforation deviations occur, the buccal flap can be performed at any time to offer a buccal view. In case of doubt, the institution of reconstructive maneuvers or even the installation of a zygomatic implant are still possible. In such situations, the benefit of maintaining muscle insertions is lost, however, the benefit of maintaining the buccal incision line is still maintained. Another important issue is the necessity of training of the operators to perform this technique since the detachment of the tissues is complex. Finally, if the surgeon aborts the immediate loading, all implants will have complete protection of intact mucosa and the use of dentures can be instituted. However, the use of complete denture can be impaired due to the attachment of the sutures on the vestibulum; this tissue presents a greater mobility and elasticity that could generate excessive pressure in the suture area causing dehiscence of the flap.

## Conclusions

A new possibility was presented with a modified access to the maxilla. This access uses information from the software to mitigate the need for wide buccal soft tissue detachments, since all the bone access for implant installation is palatal. Maintenance of the periosteum and muscle insertions adhered to the buccal region can supposedly minimize surgical trauma, edema, healing time, and pain. Further comparative studies are needed to validate and quantify these hypotheses.
